# Supplementary material for: Vaccine Literacy and Source of Information about Vaccination among Staff of Nursing Homes: A Cross-Sectional Survey Conducted in Tuscany (Italy)
Source: Vaccines (Basel). 2022 Apr 25;10(5):682. doi: 10.3390/vaccines10050682 (PMC9144185; doi:10.3390/vaccines10050682)

Supplementary Figure S1. HLVa-IT – Functional subscale - by main source of information on vaccines and vaccinations: mean values and error bars.

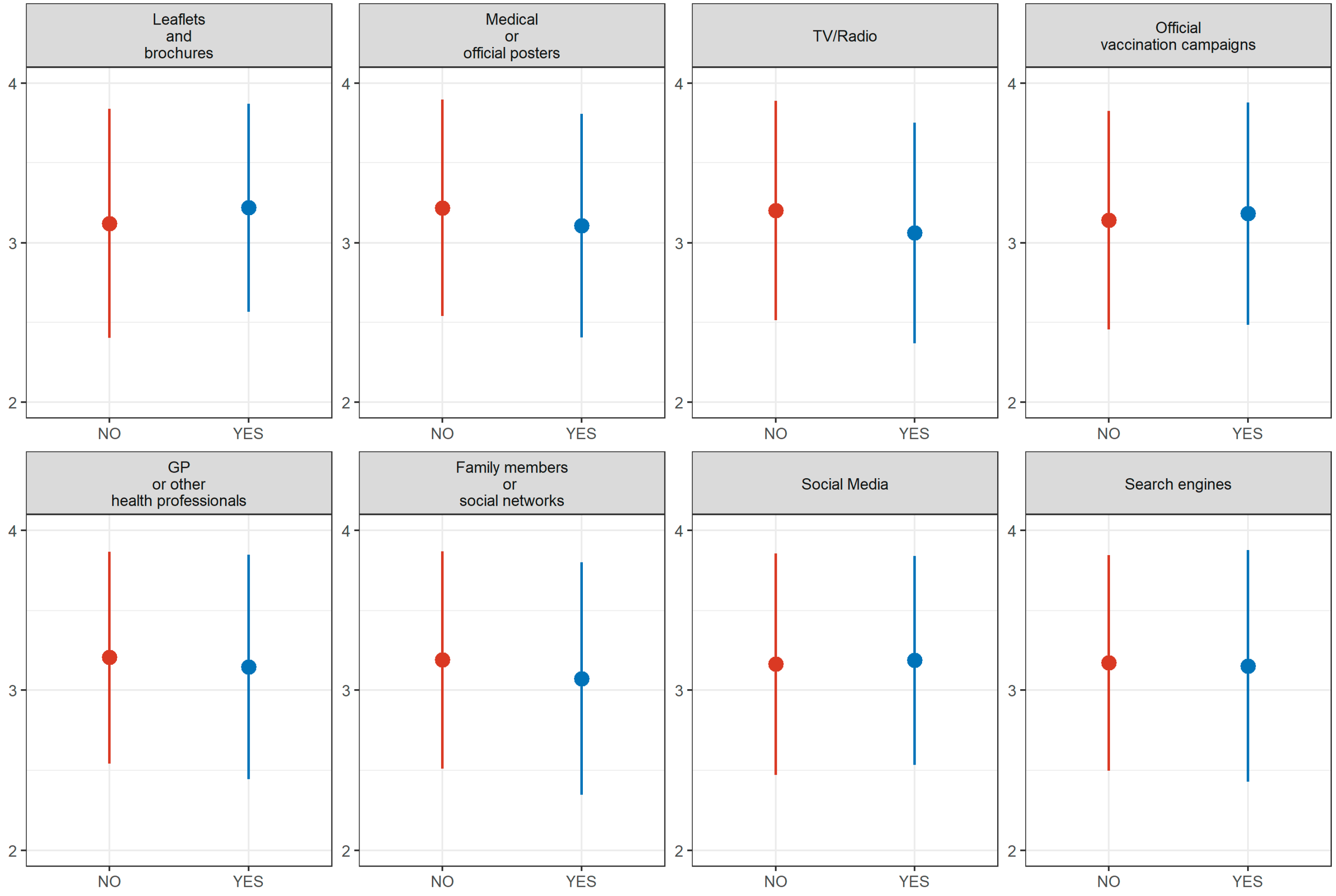

Supplementary Figure S2. HLVa-IT – interactive/communicative/critical subscale - by main source of information on vaccines and vaccinations: mean values and error bars.

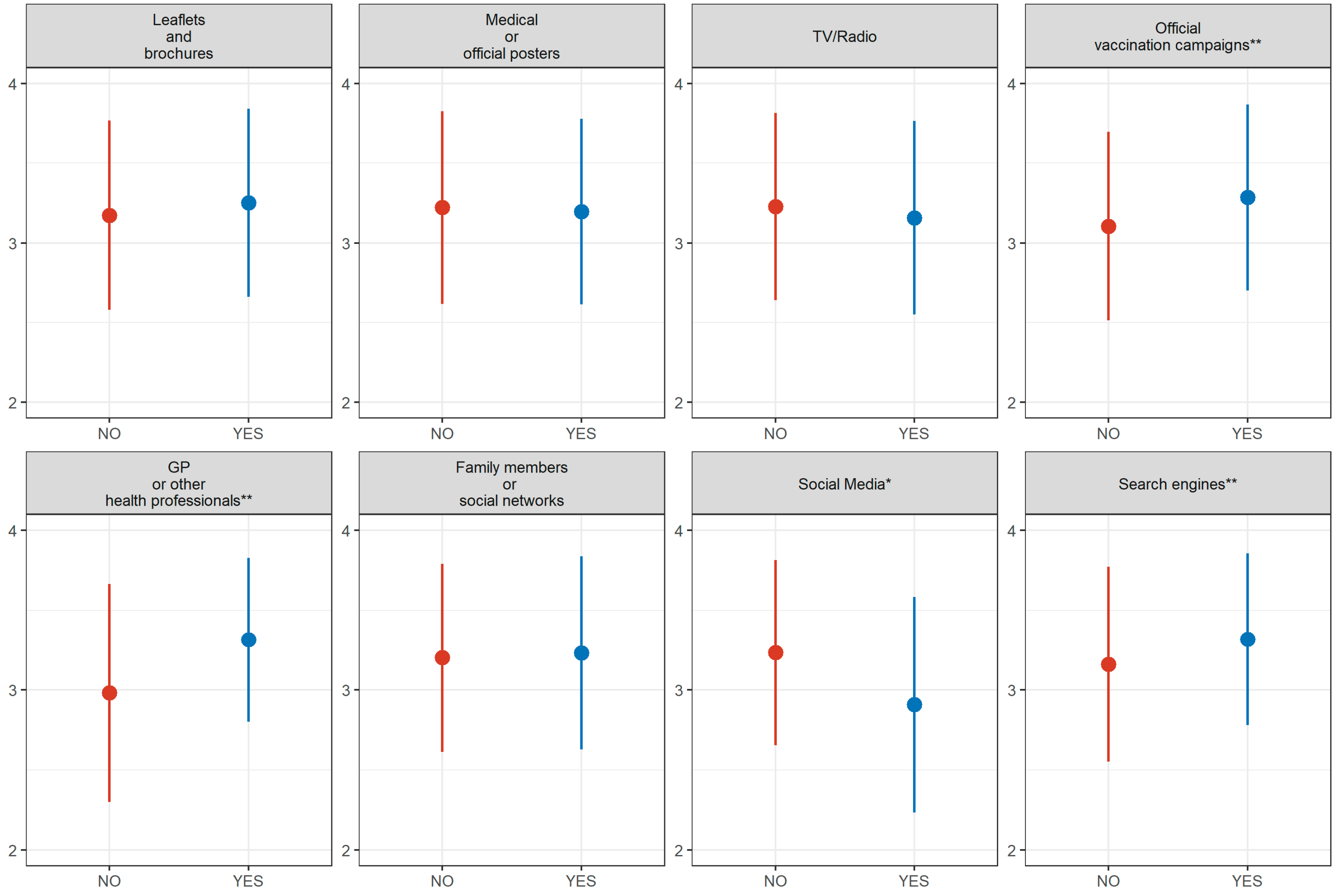

Supplementary Figure S3. HLVa-IT total score by main source of information on vaccines and vaccinations: mean values and error bars.

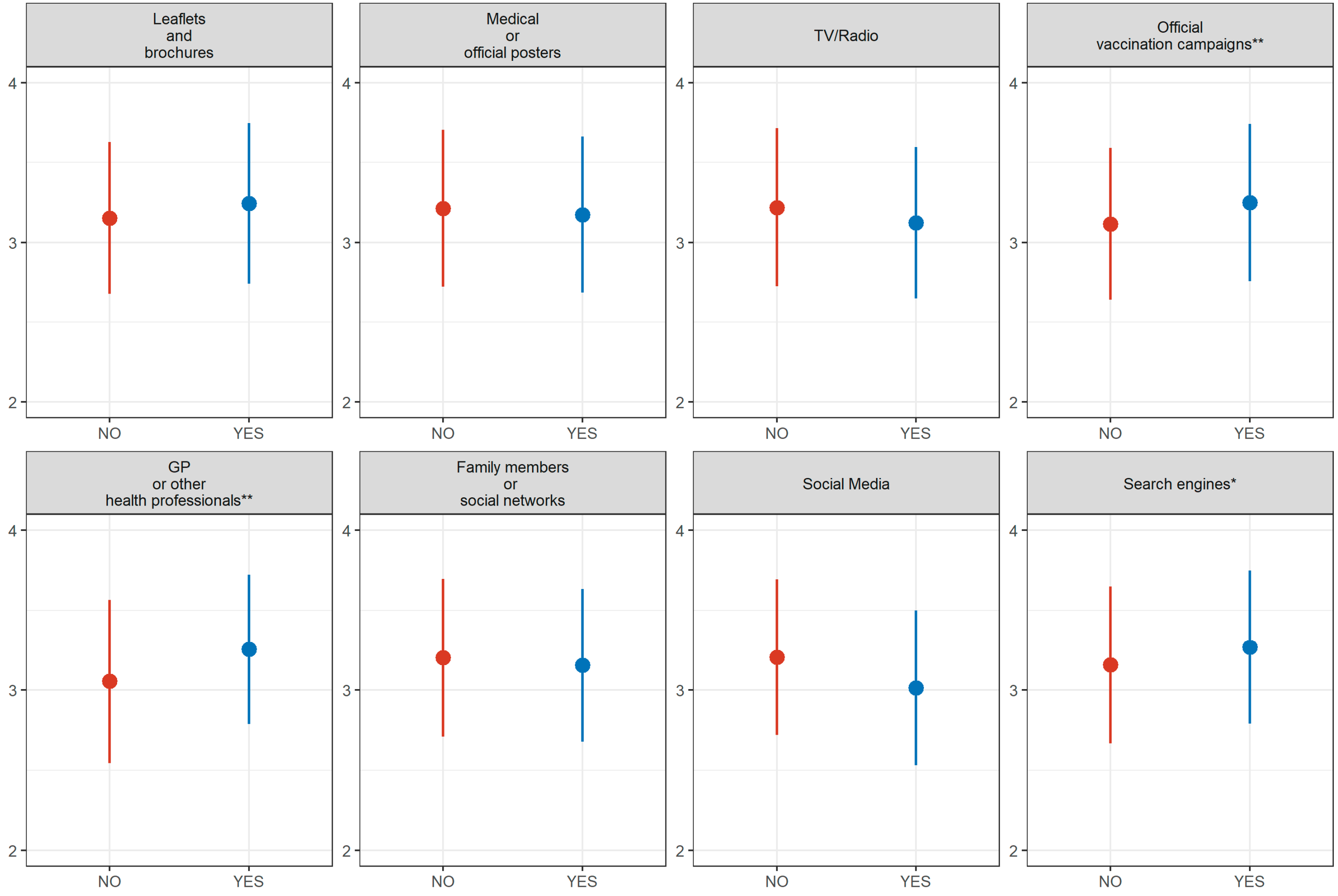

Supplement: Supplementary file 1 [file vaccines-10-00682-s001.zip › vaccines-1671869-supplementary.pdf]
